# URMC-099 facilitates amyloid-β clearance in a murine model of Alzheimer’s disease

**DOI:** 10.1186/s12974-018-1172-y

**Published:** 2018-05-05

**Authors:** Tomomi Kiyota, Jatin Machhi, Yaman Lu, Bhagyalaxmi Dyavarshetty, Maryam Nemati, Gang Zhang, R. Lee Mosley, Harris A. Gelbard, Howard E. Gendelman

**Affiliations:** 1 Department of Pharmacology and Experimental Neuroscience, University of Nebraska Medical Center, Omaha, NE USA; 2 Center for Neurotherapeutics Discovery, School of Medicine and Dentistry, University of Rochester Medical Center, Rochester, NY USA; 3 Department of Internal Medicine, University of Nebraska Medical Center, Omaha, NE USA; 4 Department of Pharmacology and Experimental Neuroscience, 985880 Nebraska Medical Center, Omaha, NE 68198-5880 USA; 5 Department of Safety Assessment, Genentech Inc., South San Francisco, CA USA; 6 Department of Pediatrics, University of California San Diego, La Jolla, CA USA

## Abstract

**Background:**

The mixed lineage kinase type 3 inhibitor URMC-099 facilitates amyloid-beta (Aβ) clearance and degradation in cultured murine microglia. One putative mechanism is an effect of URMC-099 on Aβ uptake and degradation. As URMC-099 promotes endolysosomal protein trafficking and reduces Aβ microglial pro-inflammatory activities, we assessed whether these responses affect Aβ pathobiogenesis. To this end, URMC-099’s therapeutic potential, in Aβ precursor protein/presenilin-1 (APP/PS1) double-transgenic mice, was investigated in this model of Alzheimer’s disease (AD).

**Methods:**

Four-month-old APP/PS1 mice were administered intraperitoneal URMC-099 injections at 10 mg/kg daily for 3 weeks. Brain tissues were examined by biochemical, molecular and immunohistochemical tests.

**Results:**

URMC-099 inhibited mitogen-activated protein kinase 3/4-mediated activation and attenuated β-amyloidosis. Microglial nitric oxide synthase-2 and arginase-1 were co-localized with lysosomal-associated membrane protein 1 (Lamp1) and Aβ. Importatly, URMC-099 restored synaptic integrity and hippocampal neurogenesis in APP/PS1 mice.

**Conclusions:**

URMC-099 facilitates Aβ clearance in the brain of APP/PS1 mice. The multifaceted immune modulatory and neuroprotective roles of URMC-099 make it an attractive candidate for ameliorating the course of AD. This is buttressed by removal of pathologic Aβ species and restoration of the brain’s microenvironment during disease.

## Highlights

- The therapeutic potential of the MLK3 inhibitor URMC-099 was evaluated in an AD mouse model.
- URMC-099 facilitates Aβ clearance and microglial morphological changes in diseased brain tissue.
- URMC-099 restores synaptic integrity and facilitates hippocampal neurogenesis in APP/PS1 mice.
- The multifaceted roles of URMC-099 make it an attractive therapeutic candidate for AD.

## Background

Available Alzheimer’s disease (AD) drug therapies can only transiently improve memory loss and cognitive function. All fail to restore the patient’s cognitive functions to premorbid states. Treatment options are symptomatic, designed to balance neurotransmitter and cell signaling activities, thus serving to optimize neurotransmission [1–5]. No available treatments can alter the underlying disease process or halt disease progression from mild cognitive impairment to frank dementia. These include recent trials of agents that affect amyloid-β (Aβ) clearance [6, 7]. Indeed, the recent EXPEDITION3 trial performed with solanezumab failed to meet either primary or secondary endpoints failing to slow cognitive decline (http://www.medscape.com/viewarticle/873143). Failures to affect co-morbid tauopathies [8, 9] may be yet another obstacle toward therapeutic success.

Accumulating evidence suggests that control of chronic microglial inflammation can provide an effective means to combat AD with a disease-modifying outcome. What is needed rests in developing small molecules that can both attenuate microglial inflammation while combating other disease events [10–12]. The brain-penetrant small molecule URMC-099, a mixed lineage kinase type 3 (MLK3) inhibitor, was chosen for investigation because of its control of kinase hubs responsible for inflammation and trophic activities. These qualities could ameliorate AD signs and symptoms.

The mitogen-activated protein kinase (MAPK) pathway extends from the plasma membrane to the nucleus and transmits extracellular signals from cell membrane to the intracellular targets through the network of interacting proteins [13]. MAPK subfamilies including extracellular signal-regulated kinases (ERKs), p38, and c-Jun amino-terminal kinase (JNK) play a pivotal role in cell proliferation and differentiation. The p38/JNK MAPK cascade is activated in response to toxic stimuli including pro-inflammatory mediators that promote Aβ production. MLKs belong to the MAPKKK superfamily controlling the JNK and p38 MAPK signaling cascades to transduce different immune responses including those operative in AD. MLK3, one of the most widely expressed members of the MLK family, is expressed in microglia, where it functions as an upstream inhibitor of the MAPK signaling pathway and as such could affect Aβ neurotoxicity [14, 15]. A small molecule that could affect this pathway for the treatment of AD is URMC-099 [16, 17]. Indeed, we recently demonstrated that URMC-099 facilitates Aβ clearance and degradation in cultured murine microglia by promoting Aβ uptake and degradation in endolysosomes along with attenuating microglial inflammation after Aβ exposures [18]. Based on these findings, we now posit that URMC-099 could specifically affect specific Aβ species engaged in disease pathobiology.

To these ends, we investigated the effects of URMC-099 in an AD mouse model. URMC-099 was administered to test its effects on MAPK kinase signaling, β-amyloidosis, microglial neuroinflammatory responses, synaptic activities, and hippocampal neurogenesis. We now show that URMC-099 reduces the neurotoxic burden and pro-inflammatory effects of Aβ in Aβ precursor protein/presenilin-1 (APP/PS1) mice.

## Methods

### Transgenic mice and URMC-099 administration

Transgenic mice that overexpress human APP_695_ with the Swedish mutation (designated as the Tg2576 strain) were obtained from Drs. G. Carlson and K. Hsiao-Ashe through the Mayo Medical Venture [19]. PS1 mice overexpressing human PS1 with M146L mutation (line 6.2) were provided by Dr. K. Duff through the University of South Florida [20]. Both mice were maintained in a B6/129 hybrid background [21]. Male Tg2576 mice were crossed with female PS1 mice to generate APP/PS1 double-transgenic mice. Non-transgenic (non-Tg) and APP/PS1 double-Tg mice were developed in parallel as described previously [21–23]. URMC-099 was administered as described [16, 18, 24] to 4-month-old mice that received URMC-099 by daily intraperitoneal (i.p.) injections for 3 weeks at a dose of 10 mg/kg in sterile endotoxin-free 55% saline/40% polyethylene glycol 400 (PEG400; 91893-250-F; Sigma Chemical Co., St. Louis, MO, USA)/5% dimethyl sulfoxide vehicle (Sigma) with a 27-gauge needle affixed to a sterile tuberculin syringe. This administration strategy was based in part on our previous finding that URMC-099 demonstrated its protective effect in the models of non-alcoholic steatohepatitis and human immunodeficiency virus (HIV)-associated neurocognitive disorders (HAND) at the dose of 10 mg/kg, i.p. when given for the period of 2–4 weeks [16, 25]. Mice were deeply euthanized with isoflurane, followed by blood collection then transcardially perfused with 25 ml of ice-cold phosphate-buffered saline (PBS). All animal studies adhered to the guidelines established by the Institutional Animal Care and Use Committee at University of Nebraska Medical Center.

### Tissue preparation

After transcardial perfusion, the brains were rapidly removed. The left hemisphere was dissected and immediately frozen on dry ice for biochemical testing. The right hemisphere was immersed in freshly depolymerized 4% paraformaldehyde for 48 h at 4 °C and cryoprotected by successive 24-h immersions in 15 and 30% sucrose in 1× PBS. Fixed, cryopreserved brains were sectioned coronally using a Cryostat (Leica, Bannockburn, IL, USA) with sections serially collected and stored at − 80 °C for immunohistochemistry. For biochemical testing, protein extraction and immunoblot tests were performed as described [23, 26, 27]. Protein concentration was determined using the Micro BCA Protein Assay (Thermo Fisher Scientific, Waltham, MA, USA).

### Western blot analysis

For Western blot analysis, tissue proteins were incubated with β-mercaptoethanol at 100 °C for 5 min, followed by electrophoresis on sodium dodecyl sulfate-polyacrylamide gel and transferred to polyvinylidene fluoride membrane (Immobilon-P, Millipore, Billerica, MA, USA). The membranes were blocked in 5% skim milk/Tris-buffered saline-Tween 20 (TBST) and incubated with primary antibodies (Abs) to phospho-MKK3 (p-MKK3, cat. 12280S), total-MKK3 (cat. 8535S), phospho-MKK4 (p-MKK4, cat. 4514S), total-MKK4 (cat. 9152S), phospho-p38 (p-p38, cat. 9211S), total-p38 (cat. 9212S), phospho-JNK (p-JNK, cat. 4668S), total-JNK (cat. 9258S) (1:1000, Cell Signaling Technology, Denver, MA, USA), low density lipoprotein receptor-related protein 1 (LRP1) (1:500, mouse monoclonal, Millipore Sigma, MA, USA, cat. 438,192), receptor for advanced glycation end products (RAGE) (1:1000, rabbit polyclonal, Abcam, Cambridge, MA, USA, cat. ab37647), synaptophysin (1:1000, Millipore Sigma, MA, USA, cat. MAB5258), postsynaptic density 95 (PSD95, 1:1000, Abcam, Burlington, MA, USA, cat. ab18258), arginase 1 (cat. 93668S), nitric oxide synthase-2 (NOS-2, cat. 13120S) (1:300, Cell Signaling Technology, Denver, MA, USA), and β-actin (1:2000, Sigma, St. Louis, MO, USA, cat. A3854) at 4 °C overnight, followed by 60 min incubation in 5% skim milk/TBST with horseradish peroxidase-conjugated anti-rabbit, mouse, or goat secondary Abs (1:2000, Santa Cruz Biotechnology, Santa Cruz, CA, USA). Immunoreactive bands were detected using SuperSignal West Pico or Femto Chemiluminescent substrate, and images were captured using a myECL Imager (Thermo Fisher Scientific, Waltham, MA, USA). Immunoblots were quantified using ImageJ software (NIH, Bethesda, MD, USA) relative to total protein or β-actin expression.

### Enzyme-linked immunosorbent assay

Concentrations of Aβ40 and Aβ42 in the brain extracellular fractions were quantified using human Aβ40 and Aβ42 ELISA kits (cat. KHB3482 and KHB3442, respectively) according to the manufacturer’s protocols (Thermo Fisher Scientific, Waltham, MA, USA).

### Immunohistochemistry and stereological quantification

Immunohistochemistry was performed using Abs to identify pan-Aβ (rabbit polyclonal, 1:100, Life Technologies, Carlsbad, CA, cat. 715800), Iba1 (rabbit polyclonal, 1:1000, Wako, Richmond, VA, USA, cat. sc-32725), and doublecortin (Dcx, goat polyclonal, 1:500, Santa Cruz Biotechnology, Santa Cruz, CA, USA, cat. sc-8067) [28]. Immunodetection was visualized using biotin-conjugated anti-rabbit or goat IgG used as a secondary Ab, followed by a tertiary incubation with a Vectastain ABC Elite kit (Vector Laboratories, Burlingame, CA, USA). For quantification, the areas of Aβ loads, and morphology of Iba1-positive microglia, were analyzed by investigators blinded to mouse strains and treatment at 300 μm intervals in ten 30-μm coronal sections from each mouse. Five mouse brains per group were analyzed. Briefly, Aβ-stained area was calculated by Cavalieri estimator probe (grid spacing 15 μm) of Stereo Investigator system (MBF Bioscience, Williston, VT). The Iba1-occupied area was measured using ImageJ software (NIH, Bethesda, MD, USA) by converting images to the grayscale, adjusting the threshold to cover all the stained area, and finally analyzed the particles in the region of interest. Stereological counting of Iba1-positive cells was performed using a Stereo Investigator system with an optical fractionator module [28]. In brief, the system consisted of a high-sensitivity digital camera (OrcaFlash2.8, Hamamatsu C11440-10C, Hamamatsu, Japan) interfaced with a Nikon Eclipse 90i microscope (Nikon, Melville, NY, USA). Within the Stereo Investigator program, the contour in each section was delineated using a tracing function. While sections showed tissue shrinkage along the anteroposterior axis, the extent of shrinkage between different animals was similar. The dimensions for the counting frame (120 × 100 μm) and the grid size (245 × 240 μm) were set. The *z*-plane focus was adjusted at each section for clarity, and images were automatically acquired according to each setting. The data file containing slice pictures were quantified by the fractionator with marked positive cells in analyzed areas observed in each counting frame. Based on these parameters and marked cell counts, the Stereo Investigator program computed the estimated cell populations. These total markers, cell counts, and the Gunderson (*m* = 1) values were recorded for each animal and compared between groups. For detailed Iba1 cell morphology analysis, Z stack images of 0.5 μm thickness were acquired under a × 100 oil immersion objective using a Nikon Eclipse 90i microscope. Iba1 cell bodies were analyzed in two dimensions (area at its largest cross-sectional diameter) using nucleator probe of Stereo Investigator system, whereas processes were reconstructed and analyzed in three dimensions within a single section using a computer-based tracing system (Neurolucida, MBF Bioscience, Williston, VT). For tracing and reconstruction, 10–15 random cells per section were analyzed. Similarly, stereological quantification of Dcx-positive cells was counted in a blinded fashion for every eighth section through the entire anteroposterior extent of the dentate gyrus (DG) (total 12 sections per hippocampus). Counting frame (450 × 450 μm) and grid size (500 × 500 μm) were employed for these tests.

### Immunofluorescence and confocal microscopy

The brain sections were stained with Abs to Iba1 (mouse monoclonal, 1:100, Santa Cruz Biotechnology, Santa Cruz, CA, USA, cat. sc-32725), Aβ (rabbit polyclonal, 1:100, Invitrogen, Camarillo, CA, USA, cat. 715800), and lysosomal-associated membrane protein 1 (Lamp1, rat monoclonal, 1:500, Abcam, Burlington, MA, USA, cat. ab25245), followed by incubation with Alexa Fluor 488 donkey anti-rabbit IGg, Alexa Fluor 568 donkey anti-mouse IGg, and Alexa Fluor 647 goat anti-rat IGg (1:500, Thermo Fisher Scientific, Rockford, IL, USA). Sections were mounted with Vectashield-DAPI (Vector Laboratories, Burlingame, CA, USA, cat. PK-6100). Images were captured on a LSM 710 confocal microscope (Carl Zeis Microimaging Inc., Thornwood, NY, USA) and analyzed using Zen imaging software.

### Statistical analyses

All data were normally distributed and presented as mean values ± standard errors of the mean (SEM). In case of multiple mean comparisons, the data were analyzed by one-way ANOVA and Newman-Keuls post hoc using statistics software (Prism 4.0, GraphPad Software, San Diego, CA). A value of *p* < 0.05 was regarded as a significant difference.

## Results

### URMC-099 regulates the p38/JNK MAPK signaling cascade

**Fig. 1:**
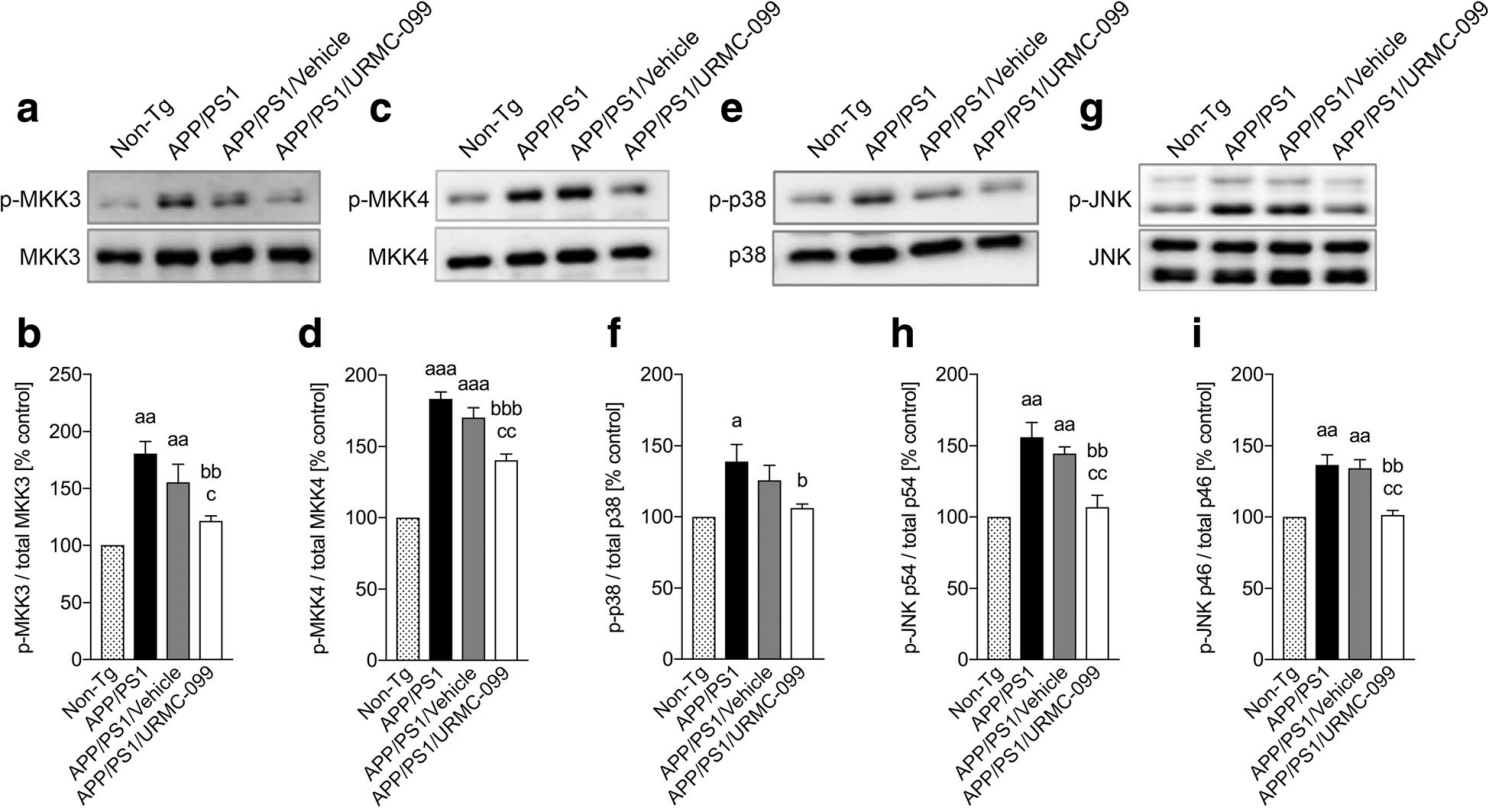
URMC-099 inhibits p38/JNK/MAPK signaling cascade in vivo. Immunoblots of p-MKK3 and total MKK3 (**a**), p-MKK4 and total MKK4 (**c**), p-p38 and total p38 (**e**), p-JNK p54 (top), p46 (bottom), and total JNK (**g**). Densitometric analysis of p-MKK3 (**b**), p-MKK4 (**d**), p-p38 (**f**), p-JNK p54 (**h**), and p46 (**i**). Data are presented as mean ± S.E.M. ^a,b,c^*p* < 0.05, ^aa,bb,cc^*p* < 0.01, ^aaa,bbb^*p* < 0.001 ^a^ vs non-Tg, ^b^ vs APP/PS1 control, ^c^ vs APP/PS1/vehicle, one-way ANOVA, and Newman-Keuls post hoc test

The brain-penetrant MLK3 inhibitor URMC-099 was previously shown to possess excellent end-organ pharmacokinetic profiles. It was first developed as an anti-inflammatory neuroprotective agent and investigated in HIV/AIDS models of human disease [16, 17, 24, 29]. We recently reported that URMC-099 attenuates microglial p38/JNK MAPK signaling cascade in vitro against Aβ toxicity. To further extend its therapeutic activities for human neurodegenerative diseases, herein, we investigated the effect of URMC-099 in APP/PS1 double-transgenic mice, a widely used AD animal model. APP/PS1 mice were treated with vehicle or URMC-099 with a single daily dose of 10 mg/kg, i.p. for 3 weeks. Non-Tg and vehicle-treated or untreated APP/PS1 mice were used as controls. Following mouse sacrifice at 5 months of age, the brains were rapidly dissected and neuropathological analyses were performed. As shown in Fig. 1, a significant increase in p-MKK3 (Fig. 1a, b, *p* < 0.01) and p-MKK4 (Fig. 1c, d, *p* < 0.001) expressions was seen in APP/PS1 and APP/PS1/vehicle groups as compared to the non-Tg control. However, URMC-099 treatment inhibited the phosphorylation of MKK3 (Fig. 1a, b, *p* < 0.01) and MKK4 (Fig. 1c, d, *p* < 0.001) in APP/PS1 mice. Additionally, we assessed the expression of phosphorylated p38/JNK, downstream regulators of MKK3/MKK4 cascade. APP/PS1 and APP/PS1/vehicle mice showed increased phosphorylation of p38 (Fig. 1e, f, *p* < 0.05), p46-JNK (Fig. 1g, i, *p* < 0.01), and p54-JNK (Fig. 1g, h, *p* < 0.01) compared to the non-Tg control. A significant reduction in phosphorylation of p38 (Fig. 1f, *p* < 0.05), p46-JNK (Fig. 1i, *p* < 0.01), and p54-JNK (Fig. 1h, *p* < 0.01) was observed with URMC-099 treatment with decreases of 23.6, 25.7, and 31.5% in p38, p46-JNK, and p54-JNK phosphorylation, respectively, as compared to the APP/PS1 group. These data further support the potential of URMC-099 to attenuate MLK3-MKK3/4-p38/JNK-mediated activation of MAPK signaling cascades in vivo.

### URMC-099 reduces β-amyloidosis in APP/PS1 mice

**Fig. 2:**
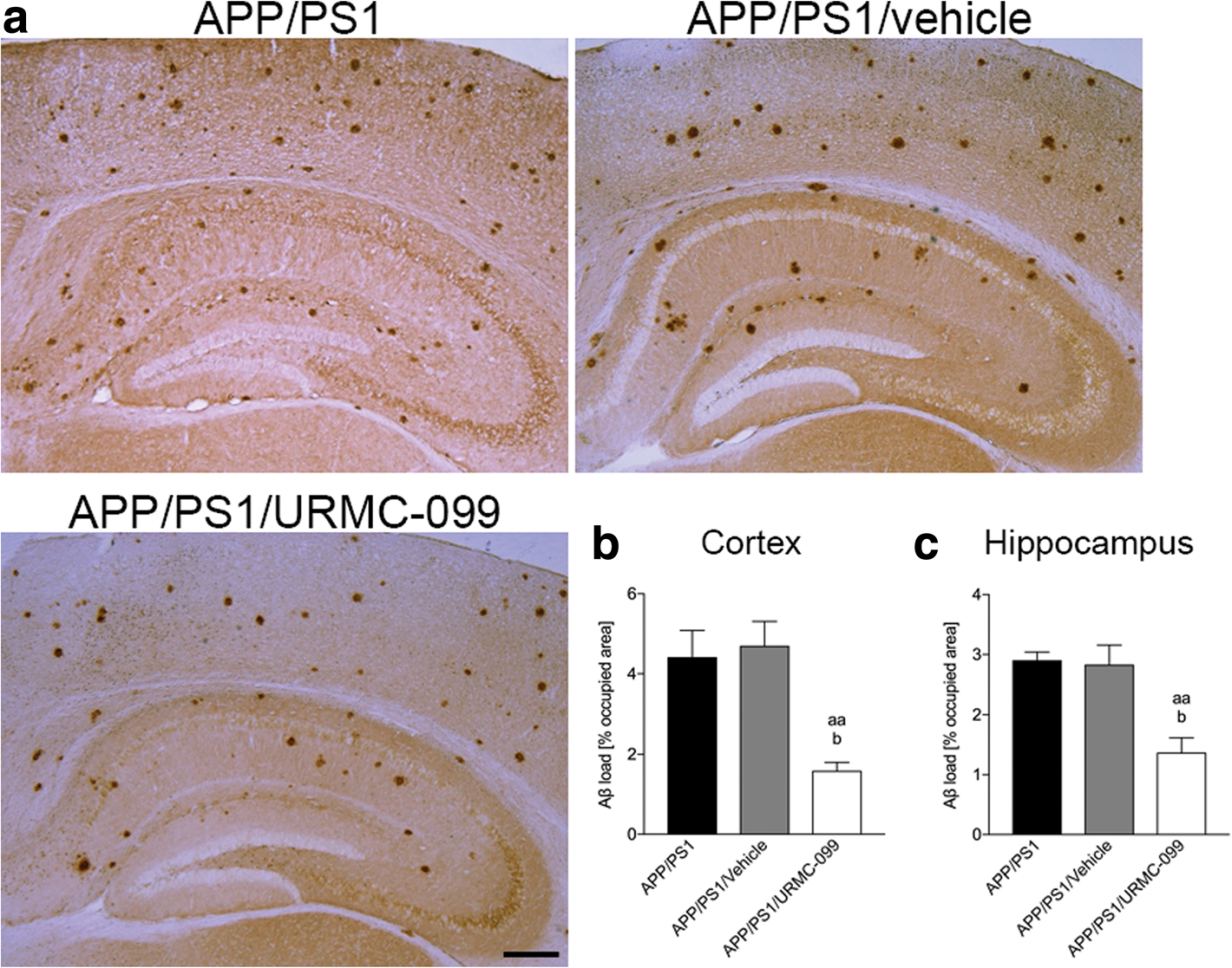
URMC-099 reduces Aβ load in the cortex and the hippocampus of APP/PS1 mice. **a** Representative images of Aβ DAB staining in the APP/PS1 mouse brain, and quantification was performed by measuring percentage-occupied area of the whole cortex (**b**) and the hippocampus (**c**), respectively. Scale bar = 500 μm (*n* = 5 per group, 12 sections per brain). Data are presented as mean ± S.E.M. ^b^*p* < 0.05, ^aa^*p* < 0.01, ^a^ vs APP/PS1 control, ^b^ vs APP/PS1/vehicle, one-way ANOVA, and Newman-Keuls post hoc test

While a beneficial role for URMC-099 in Aβ clearance and inflammation was shown in cultured microglia, the drug's action(s) in animal AD models was not previously investigated [18]. Thus, we examined the effects of URMC-099 on β-amyloidosis in the cortex and the hippocampus in APP/PS1 mice (Fig. 2). Total Aβ load, composed of diffuse and compact plaques, was determined by Aβ DAB stainings (Fig. 2a). In URMC-099-treated animals, the total Aβ load was reduced both in the cortex [64.1% (Fig. 2b, *p* < 0.01) and 66.2% (Fig. 2b, *p* < 0.05) of treated compared to APP/PS1 and APP/PS1/vehicle groups, respectively] and the hippocampus [52.8% (Fig. 2c, *p* < 0.01) and 51.6% (Fig. 2c, *p* < 0.05) of treated compared to APP/PS1 and APP/PS1/vehicle groups, respectively]. These results reveal the ability of URMC-099 treatment to significantly ameliorate the central nervous system (CNS) burden of β-amyloidosis in APP/PS1 mice.

**Fig. 3:**
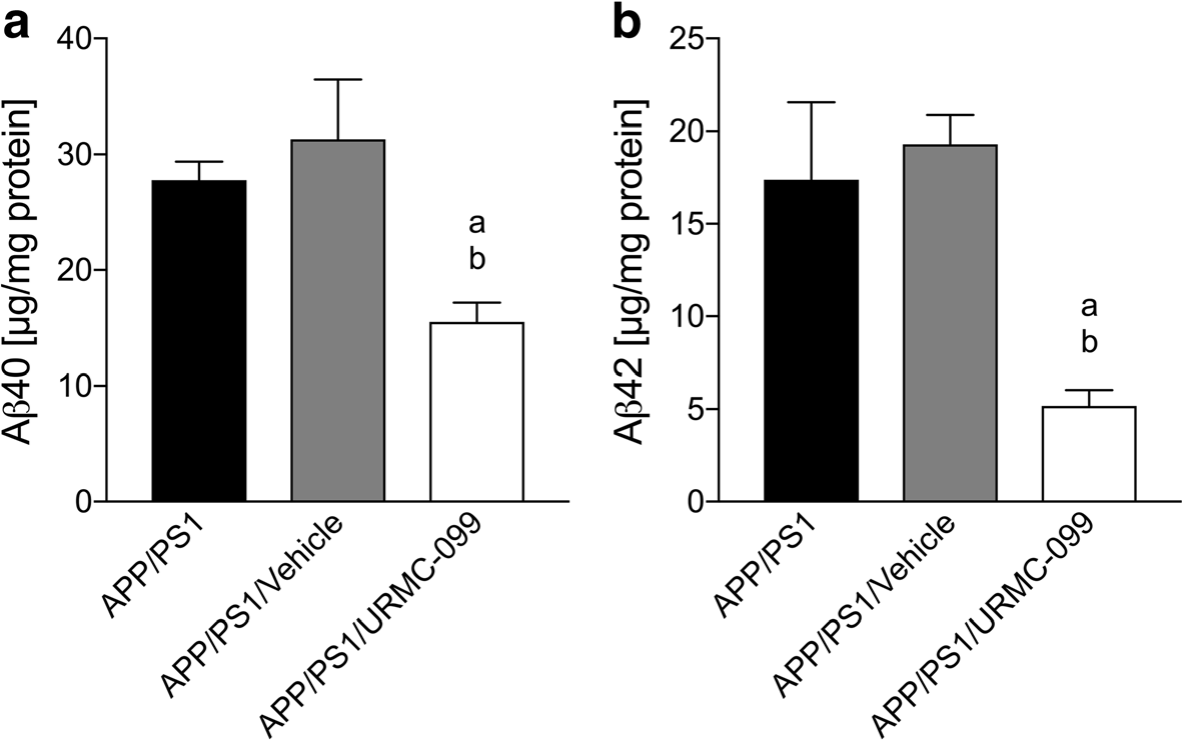
URMC-099 reduces extracellular Aβ40 and Aβ42 levels in the APP/PS1 brain. The levels of Aβ40 (**a**) and Aβ42 (**b**) in an extracellular-enriched fraction were measured by human Aβ40- and Aβ42-specific ELISAs. Data are presented as mean ± S.E.M. ^a,b^*p* < 0.05, ^a^ vs APP/PS1 control, ^b^ vs APP/PS1/vehicle, one-way ANOVA, and Newman-Keuls post hoc test

To gain a better understanding of how URMC-099 affects of β-amyloidosis, we assessed Aβ40 and Aβ42 levels in the APP/PS1 mouse brain. The extracellular brain fractions were subjected to Aβ ELISA tests (Fig. 3). Both Aβ40 and Aβ42 levels were decreased in the brain after URMC-099 treatment [55.9 and 50.0% for Aβ40 (Fig. 3a, *p* < 0.05), 30.0 and 26.9% for Aβ42 (Fig. 3b, *p* < 0.05), as compared to APP/PS1 and APP/PS1/vehicle groups, respectively], raising the possibility that Aβ species may have increased clearance from the CNS in URMC-099-treated APP/PS1 mice.

### URMC-099 modifies Aβ transporters

**Fig. 4:**
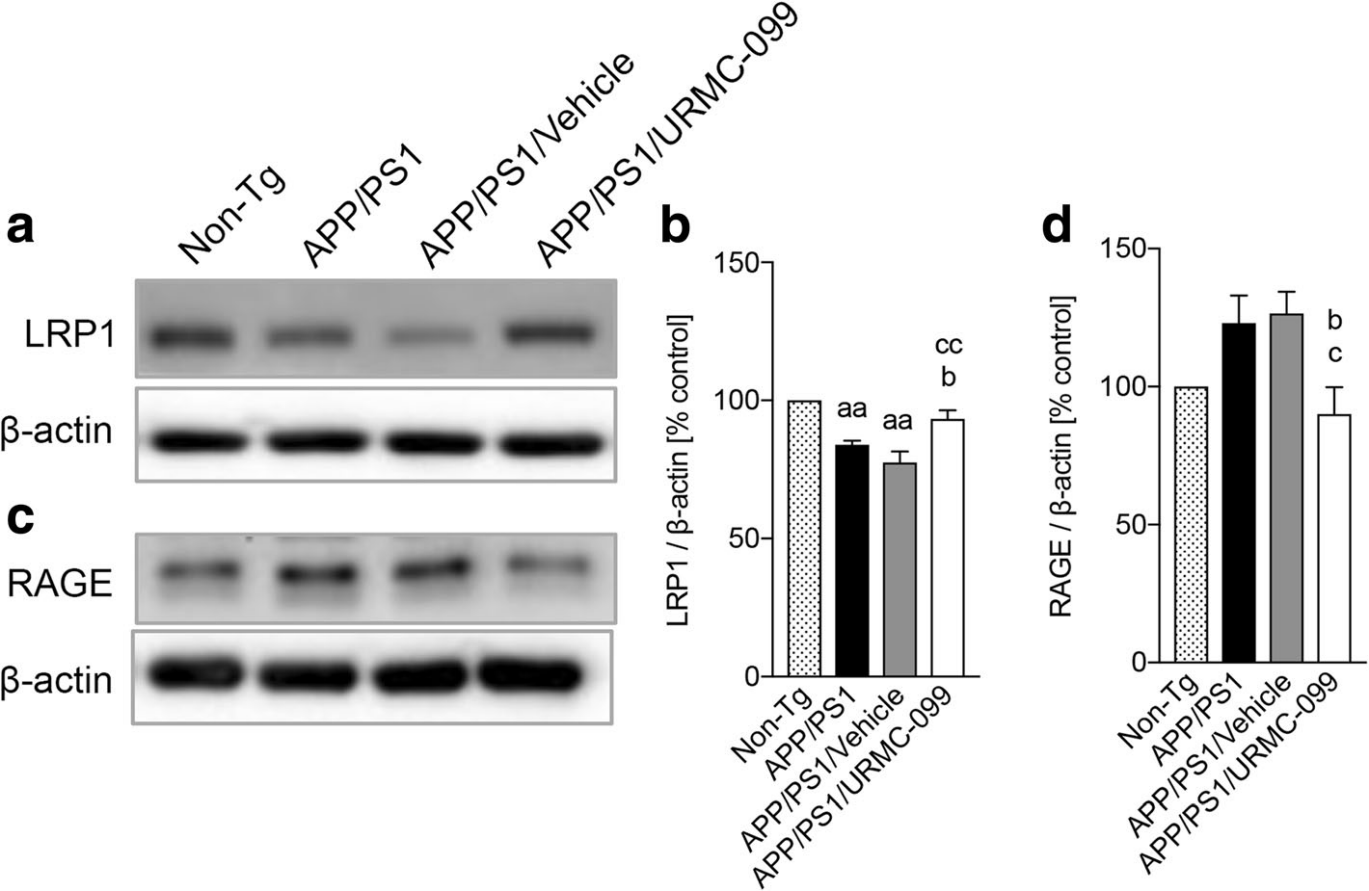
URMC-099 modulates Aβ transporter levels in the APP/PS1 brain. Immunoblots of LRP1 (**a**) and RAGE (**c**). Densitometric analysis of LRP1 (**b**) and RAGE (**d**). Data are presented as mean ± S.E.M. ^b,c^*p* < 0.05, ^aa,cc^*p* < 0.01, ^a^ vs non-Tg, ^b^ vs APP/PS1 control, ^c^ vs APP/PS1/vehicle, one-way ANOVA, and Newman-Keuls post hoc test

The Aβ brain levels are attributed to transporters located in the blood-brain barrier (BBB) [30, 31]. The ability of URMC-099 to affect Aβ brain clearance was assessed by determining LRP1 and RAGE expression levels, an Aβ efflux conducting and influx receptor, respectively, in the brain (Fig. 4a–c). LRP1 expression was significantly reduced in APP/PS1 (Fig. 4b, *p* < 0.01) and APP/PS1/vehicle (Fig. 4a, b, *p* < 0.01) groups as compared to the non-Tg controls. URMC-099 treatment reversed these outcomes (Fig. 4b *p* < 0.05). Contrary to these results, RAGE levels were significantly reduced in URMC-099-treated APP/PS1 mice compared to non-treated or vehicle-treated APP/PS1 mice (Fig. 4c, d *p* < 0.05). Aβ influx into the brain was decreased in APP/PS1 mice that received URMC-099. Thus, URMC-099 facilitate Aβ clearance from the brain.

### URMC-099 changes microglial morphology in the brain of APP/PS1 mice

**Fig. 5:**
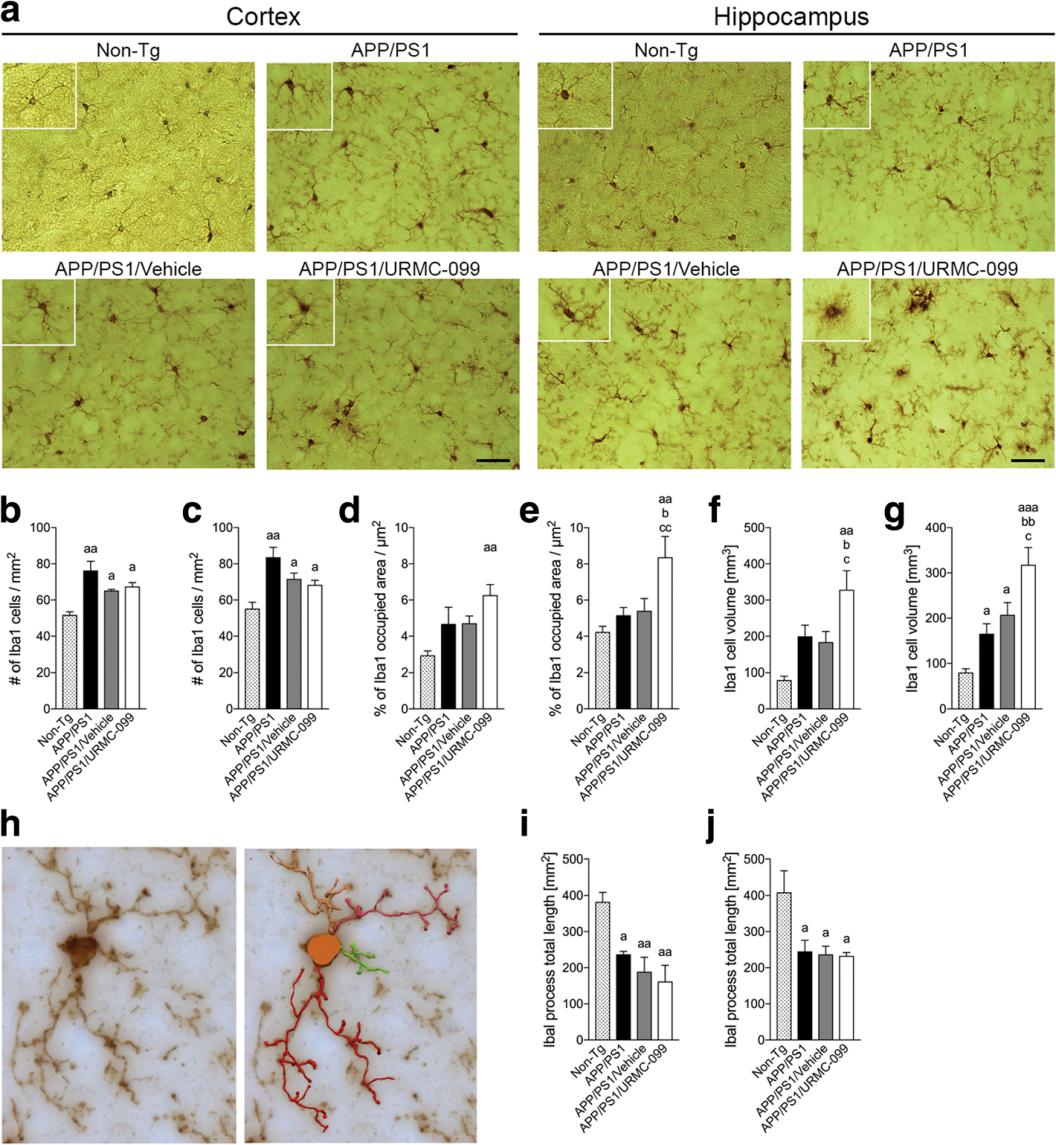
URMC-099 alters microglial morphology in APP/PS1 mice. **a** Representative images of Iba1 staining in the cortex and the hippocampus of APP/PS1 mice are shown. Scale bar = 50 μm. **b**, **c** Quantification of total Iba1 cell count in the cortex (**b**) and the hippocampus (**c**) of the brain. **d**, **e** Quantification of the area of Iba1-positive cells including cell processes per area (μm^2^) in the cortex (**d**) and the hippocampus (**e**) (*n* = 5 per group, 12 sections per brain, three areas/section) are shown. **f**, **g** Quantification of Iba1 cell body volume in the cortex (**f**) and the hippocampus (**g**) of the brain. **h** Representative reconstruction of Iba1 cell process in three dimensions. **i**, **j** Quantification of Iba1 cell process length in the cortex (**i**) and the hippocampus (**j**). Data are presented as mean ± S.E.M. ^a,b,c^*p* < 0.05, ^aa,bb,cc^*p* < 0.01, ^aaa^*p* < 0.001, ^a^ vs non-Tg, ^b^ vs APP/PS1, ^c^ vs APP/PS1/vehicle, one-way ANOVA, and Newman-Keuls post hoc test

URMC-099 treatment has been previously shown to affect microglial morphology associated with an inflammatory phenotype after exposure to the HIV-1 Tat protein [16]. This is considered to be a neuroprotective feature of URMC-099. To investigate if URMC-099 similarly reverses pathologic microglial morphology present in APP/PS1 mice, number and area of Iba1-positive microglia were quantified (Fig. 5). In both the cortex and hippocampus, numbers of Iba1-positive cells were increased in treated or untreated APP/PS1 mice compared to non-Tg mice (Fig. 5a–c). Iba1-positive tissue areas were quantified. In the cortex (Fig. 5d), untreated or vehicle-treated APP/PS1 mice showed increases in microglial staining areas. The Iba1 brain areas were observed to be greater in URMC-099-treated APP/PS1 mice compared to non-Tg controls (Fig. 5d, *p* < 0.01). In the hippocampus (Fig. 5e), similar results were observed as shown in the cortex (Fig. 5e, *p* < 0.01 to non-Tg and vehicle-treated APP/PS1, *p* < 0.05 to untreated APP/PS1. To best assess Iba1 cell morphology, the cell body volume and cell process lengths were quantified. In the cortex (Fig. 5f), the volume was increased in all APP/PS1 mouse groups as compared to non-Tg mice. Moreover, URMC-099 treatment further increased cell body volume with a significant difference (Fig. 5f, *p* < 0.01 to non-Tg, *p* < 0.05 to untreated or vehicle-injected APP/PS1). In the hippocampus (Fig. 5g), similar results were obtained with the URMC-099 treatment, which increased cell body volume as compared to other groups (Fig. 5g, *p* < 0.001 to non-Tg control, *p* < 0.01 to untreated, and *p* < 0.05 to vehicle-treated APP/PS1 mice), supported by our previous findings showing increased phagocytic microglia with URMC-099 treatment [18]. Cell process length was analyzed by reconstructing individual cell in three dimensions (Fig. 5h). As discussed earlier, non-Tg animals displayed least cell body volume with small spherical shape (see Fig. 5a). Therefore, resembling to the resting or ramified phenotype, Iba1-positive cells in non-Tg mice showed the longest process length compared to treated or untreated APP/PS1 mice both in the cortex (Fig. 5i, *p* < 0.01) and the hippocampus (Fig. 5j, *p* < 0.05). With increased cell body volume, Iba1-positive cells showed a decrease in cell body roundness and progress more to an amoeboid shape associated with reduced ramification (see Fig. 5a). Here, no significant difference was observed in process length among all the treated or untreated APP/PS1 mice both in the cortex (Fig. 5i) and the hippocampus (Fig. 5j). Overall, the data validates the findings that URMC-099 modifies microglial morphologies in APP/PS1 mice.

### URMC-099 facilitates M2 microglia phenotype polarization

**Fig. 6:**
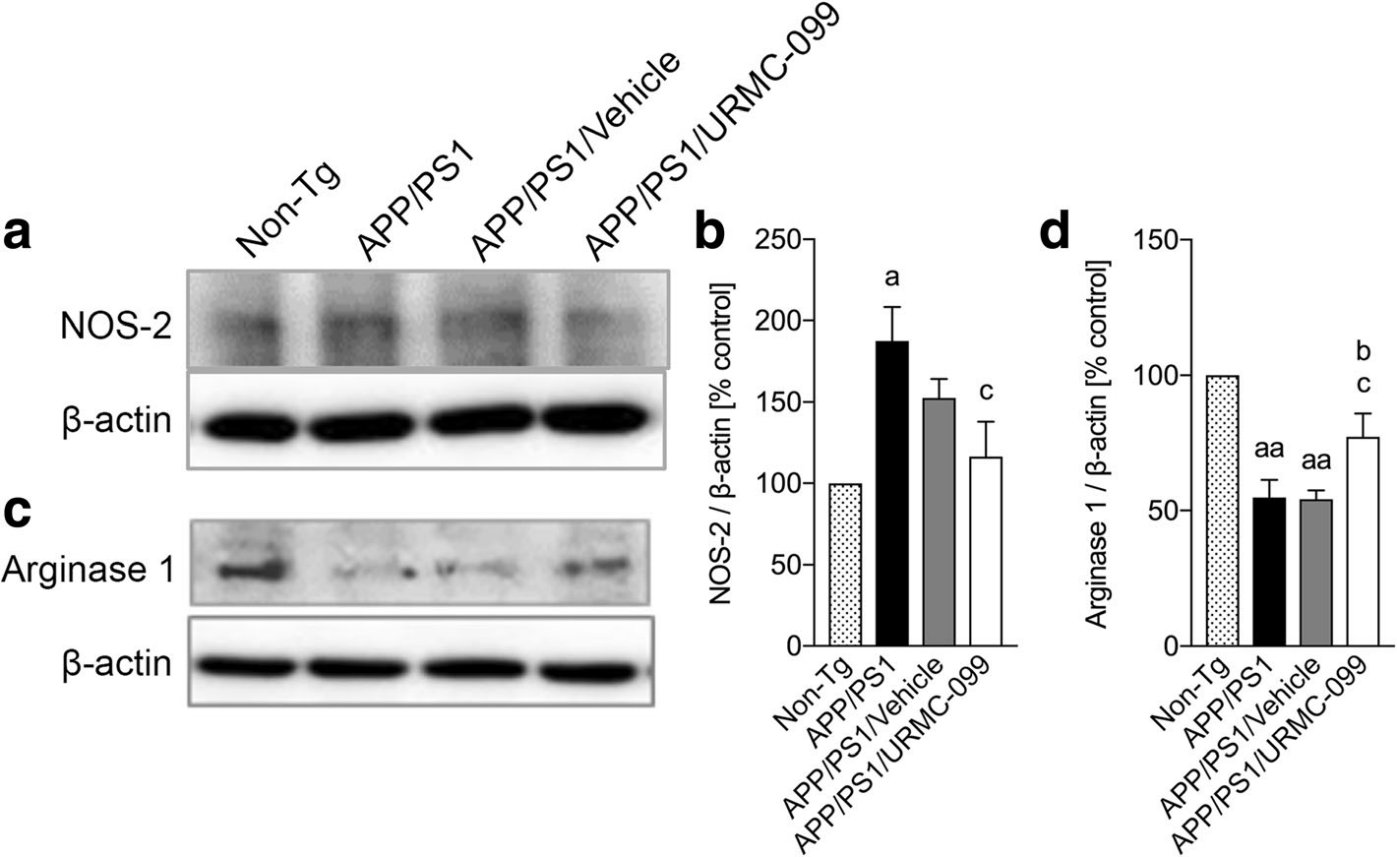
URMC-099 facilitates microglial M2 phenotype polarization. Immunoblots of NOS-2 (**a**) and arginase 1 (**c**). Densitometric analysis of NOS-2 (**b**) and arginase 1 (**d**). Data are presented as mean ± S.E.M. ^a,b,c^*p* < 0.05, ^aa^*p* < 0.01, ^a^ vs non-Tg, ^b^ vs APP/PS1 control, ^c^ vs APP/PS1/vehicle one-way ANOVA, and Newman-Keuls post hoc test

Microglia are a unique population of innate immune cells in the CNS having the ability to polarize into two different phenotypes: M1 (classical activation) and M2 (alternative activation) that can directly affect synaptic communication. M1 microglia are pro-inflammatory while M2 microglia are anti-inflammatory [32, 33]. A drug that is effective in altering microglial responses to neurotoxic stimuli with a shift from an M1 to a M2 phenotype can attenuate pro-inflammatory innate immune responses that can exacerbate bystander damage to normal synaptic architecture and thus could be a candidate for human therapeutic intervention in this aspect of AD neuropathogenesis. To address the role of microglial phenotypes in APP/PS1 mice, Western blot analysis assessed the relative expression of NOS-2 as a correlate of M1 response and arginase 1 as a correlate of M2 response, respectively (Fig. 6a–d). The APP/PS1 control group showed increased expression of NOS-2 as compared to the non-Tg control group (Fig. 6a, b, *p* < 0.05). In contrast, arginase 1 levels were reduced in vehicle-treated or untreated APP/PS1 mice (Fig. 6c, d, *p* < 0.01) as compared to the non-Tg mice, suggesting an increased M1 pro-inflammatory phenotype and a reduced M2 anti-inflammatory phenotype microglia in APP/PS1 and APP/PS1/vehicle groups. However, URMC-099 treatment ameliorated NOS-2 expression (Fig. 6b, *p* < 0.05) and increased arginase 1 levels (Fig. 6d, *p* < 0.05) in APP/PS1 mouse brain, demonstrating URMC-099’s ability to decrease the M1 phenotype so that the M2 phenotype could ameliorate Aβ neurotoxicity.

### URMC-099 increases co-localization of Aβ with Lamp1 in microglia

**Fig. 7:**
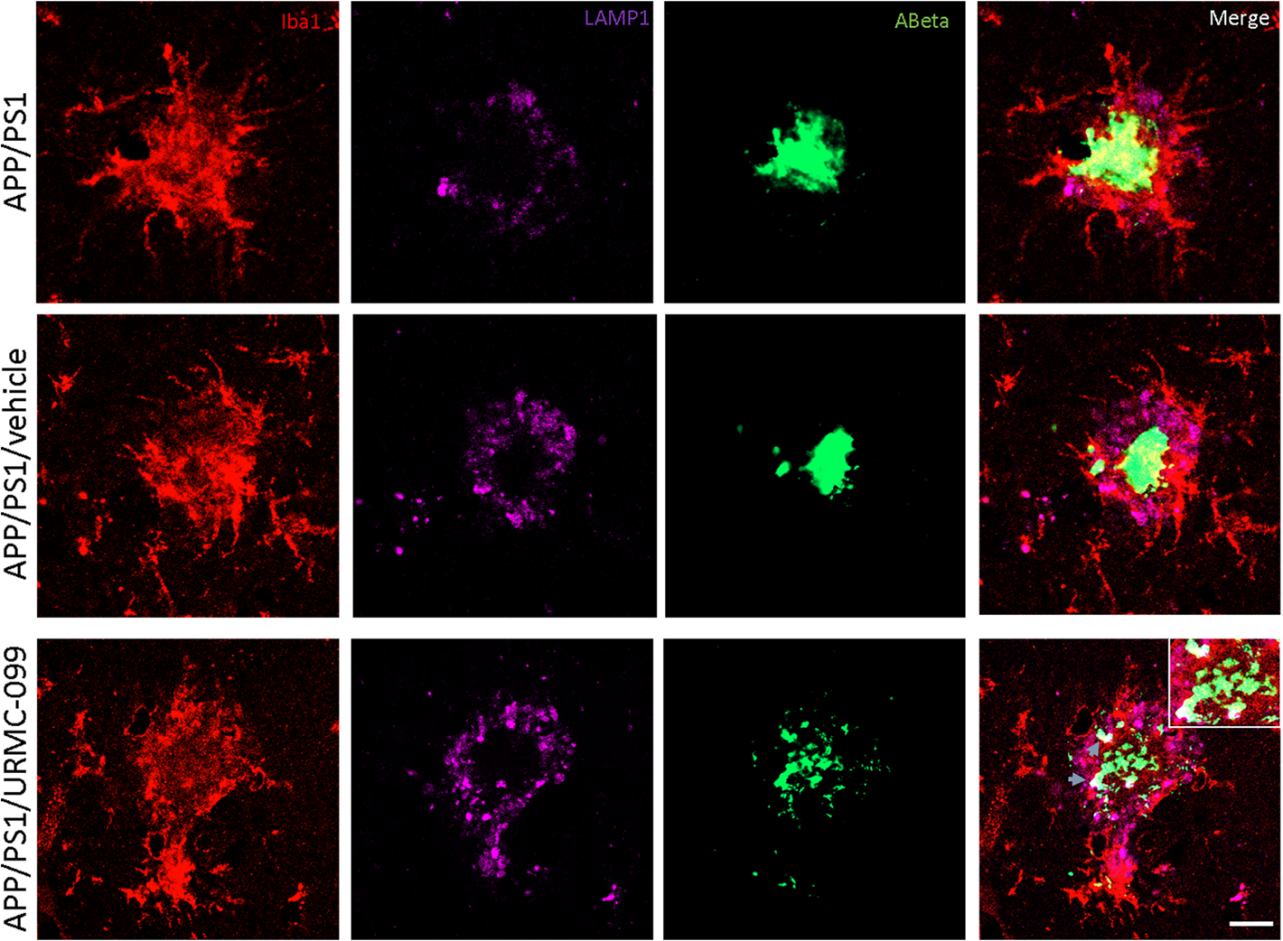
URMC-099 increases co-localization of Aβ with LAMP1 in microglia. APP/PS1 mouse brain slices were immunohistochemically labeled for Iba1 (red), Aβ plaques (green), and LAMP1 (magenta). Co-localization of Aβ with LAMP1 in microglia is represented by overlay of three channels. The box indicates the most prominent co-localization of Aβ and LAMP1. Scale bar = 5 μm

Aβ has the ability to bind with the scavenger receptors present on microglia, followed by the phagocytic engulfment of Aβ. This phagocytized Aβ then enters into the endolysosomal degradation pathway of microglia in response to extracellular accumulation of Aβ [18]. Thus, we investigated the effect of URMC-099 on the endolysosomal degradation of Aβ in microglia in vivo. Confocal microscopy showed increased accumulation of microglia surrounding Aβ in all the experimental groups. However, co-localization of Aβ with Lamp1 increased in the APP/PS1 URMC-099-treated group (Fig. 7, indicated by arrow), suggesting enhanced lysosomal Aβ degradation. No co-localization of Aβ with Lamp1 was observed in the APP/PS1 untreated or APP/PS1/vehicle groups. The results suggest that URMC-099 treatment can promote microglial endolysosomal degradation of Aβ in vivo.

### URMC-099 restores postsynaptic markers

**Fig. 8:**
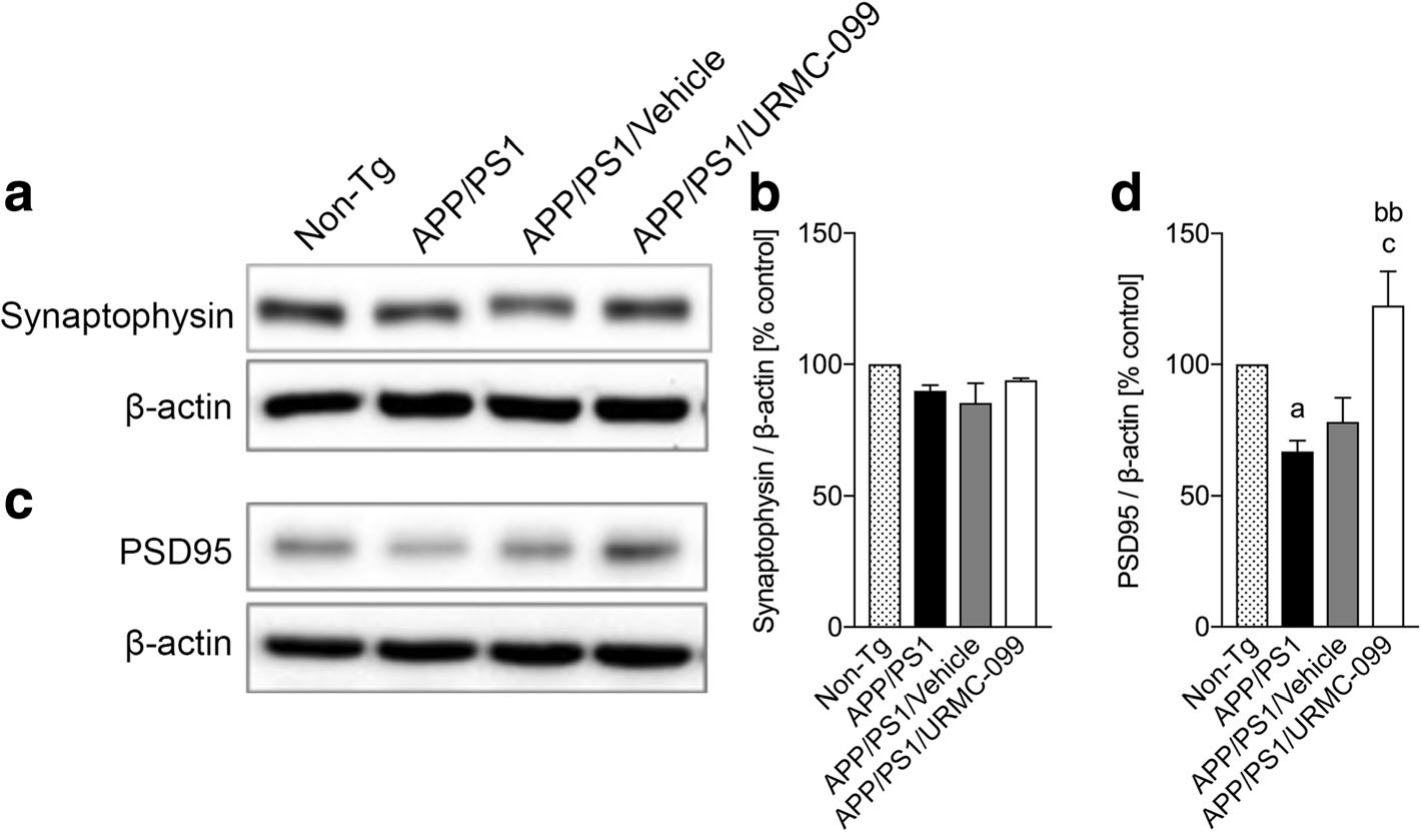
URMC-099 restores synaptic integrity. Immunoblots of synaptophysin (presynaptic) (**a**) and PSD95 (postsynaptic) (**c**). Densitometric analysis of synaptophysin (**b**) and PSD95 (**d**). Data are presented as mean ± S.E.M. ^a,c^*p* < 0.05, ^bb^*p* < 0.01, ^a^ vs non-Tg, ^b^ vs APP/PS1 control, one-way ANOVA, and Newman-Keuls post hoc test

AD is characterized by cognitive impairment. Synaptic density correlates with memory function [34] and is estimated by synaptophysin (presynaptic marker) and postsynaptic density 95 (PSD95: postsynaptic marker) protein stains [35]. Synaptophysin and other synaptic vesicle proteins along with the proteins of postsynaptic densities such as PSD95 are linked to synaptic plasticity and learning and memory formation [36, 37]. Thus, we measured synaptophysin and PSD95 in the hippocampal region of APP/PS1 and non-Tg mice (Fig. 8). No significant changes were observed in synaptophysin expression across all experimental groups (Fig. 8b). However, PSD95 levels were reduced in APP/PS1 mice compared to non-Tg animals (Fig. 8d, *p* < 0.05). URMC-099 treatment significantly elevated PSD95 levels in AD mice compared to APP/PS1 (Fig. 8d, *p* < 0.01) and APP/PS1/vehicle (Fig. 8d, *p* < 0.05) mouse groups. Reduced postsynaptic proteins were markers of APP/PS1 and APP/PS1/vehicle mouse groups were likely linked to memory impairments. In contrast, URMC-099 can restore postsynaptic proteins that, in turn, allow for restoration of normal synaptic function.

### URMC-099 protects hippocampal neurogenesis in APP/PS1 mice

**Fig. 9:**
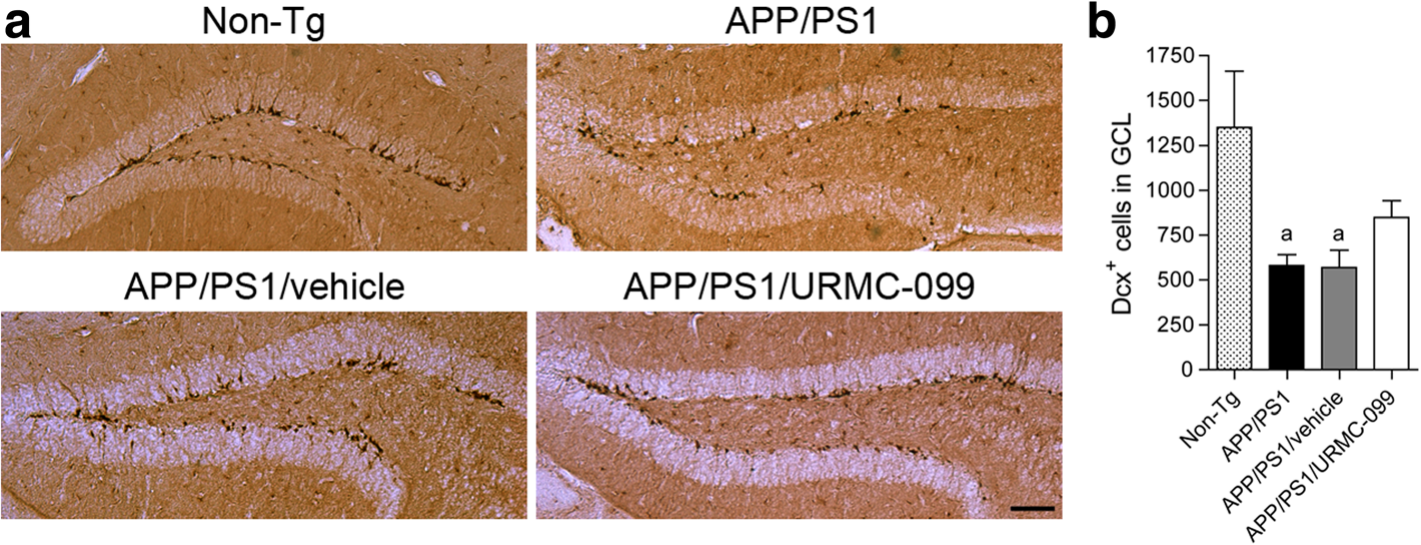
URMC-099 protects hippocampal neurogenesis in the DG of APP/PS1 mice. **a** Immunohistochemical detection of Dcx-labeled cells in the DG from 5-month-old mice are illustrated. Scale bar = 200 μm. **b** Quantification of the numbers of Dcx-labeled cells in the DG (*n* = 5 mice per group, 12 sections per mouse). Data are presented as means ± S.E.M. ^a^*p* < 0.05, ^a^ vs non-Tg control, one-way ANOVA, and Newman-Keuls post hoc test

Because URMC-099 treatment is associated with normalized levels of PSD-95, we next asked whether its potential trophic effects could extend to the restoration of neurogenesis in the DG. Therefore, we examined the expression of the microtubule-associated protein Dcx within the DG (Fig. 9a, b). Dcx is a marker for newly generated premature neurons in the subgranular zone of the DG serving as a reliable screen for neurogenesis. Notably, the numbers of Dcx^+^ cells in APP/PS1 and APP/PS1/vehicle groups were significantly decreased as compared to non-Tg mice (43 and 42.2% of non-Tg control, Fig. 9b, *p* < 0.05). However, the Dcx^+^ cell number in APP/PS1/URMC-099 mice was increased as compared to APP/PS1 and APP/PS1/vehicle groups (146 and 149% of the APP/PS1 and APP/PS1/vehicle groups, respectively, Fig. 9b). These data indicate that populations of neuronal precursors are protected or restored by URMC-099 treatment in APP/PS1 mice.

## Discussion

AD is one of the most common age-related neurodegenerative disorders and is associated with pathologic Aβ deposition, abnormal tau phosphorylation, and neuroinflammation. In the AD brain, Aβ and neurofibrillary tangles can directly cause neuronal damage and cell death. Indirectly, they can also accelerate neuronal degeneration by inducing inflammatory cytokines, chemokines, and neurotoxins through activation of the innate immune system. Otherwise, innate immunity plays a protective homeostatic role [38, 39]. Microglia and astrocytes are the main immune cells within the central nervous system. Aβ-stimulated microglial inflammatory responses engage mitogen-activated protein kinase (MAPK) and c-Jun amino-terminal kinase (JNK) signaling pathways in AD, which are modulated by URMC-099. Because its pharmacokinetic (brain-penetrant) and pharmacodynamic profiles are so favorable for the treatment of neuroinflammatory disease, it is an attractive candidate to effectively control Aβ-mediated neuroinflammation and potentially decrease or halt neurodegeneration. Additionally, URMC-099 also modulates pathways involved in Aβ trafficking and processing required for AD-associated microglial neurotoxic activities [18]. Our data in aggregate lend support to the therapeutic potential of MLK3 inhibitors by their parallel abilities to inhibit neuronal apoptosis and elicit neurotrophic responses together with reversal of pathologic microglial activation responses [14, 40, 41].

Microglia are the resident immune cells of the CNS and recognized as pivotal regulators of the brain immunity and homeostasis with important roles in different neurological disorders including AD. Microglia serve as the first line of immune defense against invading pathogens or pathologic forms of host proteins in the CNS. They initiate the innate immune response by recognizing pathogen-associated microbial patterns (PAMPs) and inducing key co-stimulatory molecules and cytokines, which induce the adaptive immune response in the diseased brain [42–44]. In the AD brain, microglia also serve as scavenger cells that phagocytose amyloid-β peptides (Aβ) [44–46]. In parallel, microglia also contribute to tissue injury due to neuroinflammation. Notably, these cells can be polarized into specific phenotypes based on their functional properties [10, 47–50]. Microglia react to misfolded Aβ by polarization into an M1 phenotype (classical activation) to exacerbate neuroinflammatory responses [10, 43, 51]. Classical activation is associated with the production of pro-inflammatory molecules such as interleukin (IL)-1β, IL-6, tumor necrosis factor (TNF)-α, neurotoxic reactive oxygen species, and nitric oxide [52–58]. However, microglia can also acquire an M2 phenotype (alternative activation) in AD to combat against neuroinflammation and pathogenic Aβ plaque deposition after the onset of classical activation [10, 11, 47, 59, 60]. M2 microglia can be neuroprotective as shown by their anti-inflammatory characteristics with the secretion of anti-inflammatory cytokines IL-4, IL-13, and transforming growth factor (TGF)-β [61–63] and increased Aβ phagocytosis and degradation without production of neurotoxins [61–64]. Thus, recent reports suggest that microglia can adopt opposing activation phenotypes based upon different microenvironment signals [59, 60]. Microglia acquire an anti-inflammatory M2 phenotype in the early phase of AD surrounding the plaques for Aβ phagocytosis and degradation but most likely shift into the pro-inflammatory M1 state with disease progression. Thus, a balance between M1 and M2 phenotypes seems to be disturbed with a pathologic increase in the M1 phenotype that accompanies disease progression [47]. Hence, restoration of the balance between M1/M2 phenotypes is one of the ideal therapeutic strategies for treating AD. Such a restorative function is indicative of what was observed by URMC-099 in the current report.

**Fig. 10:**
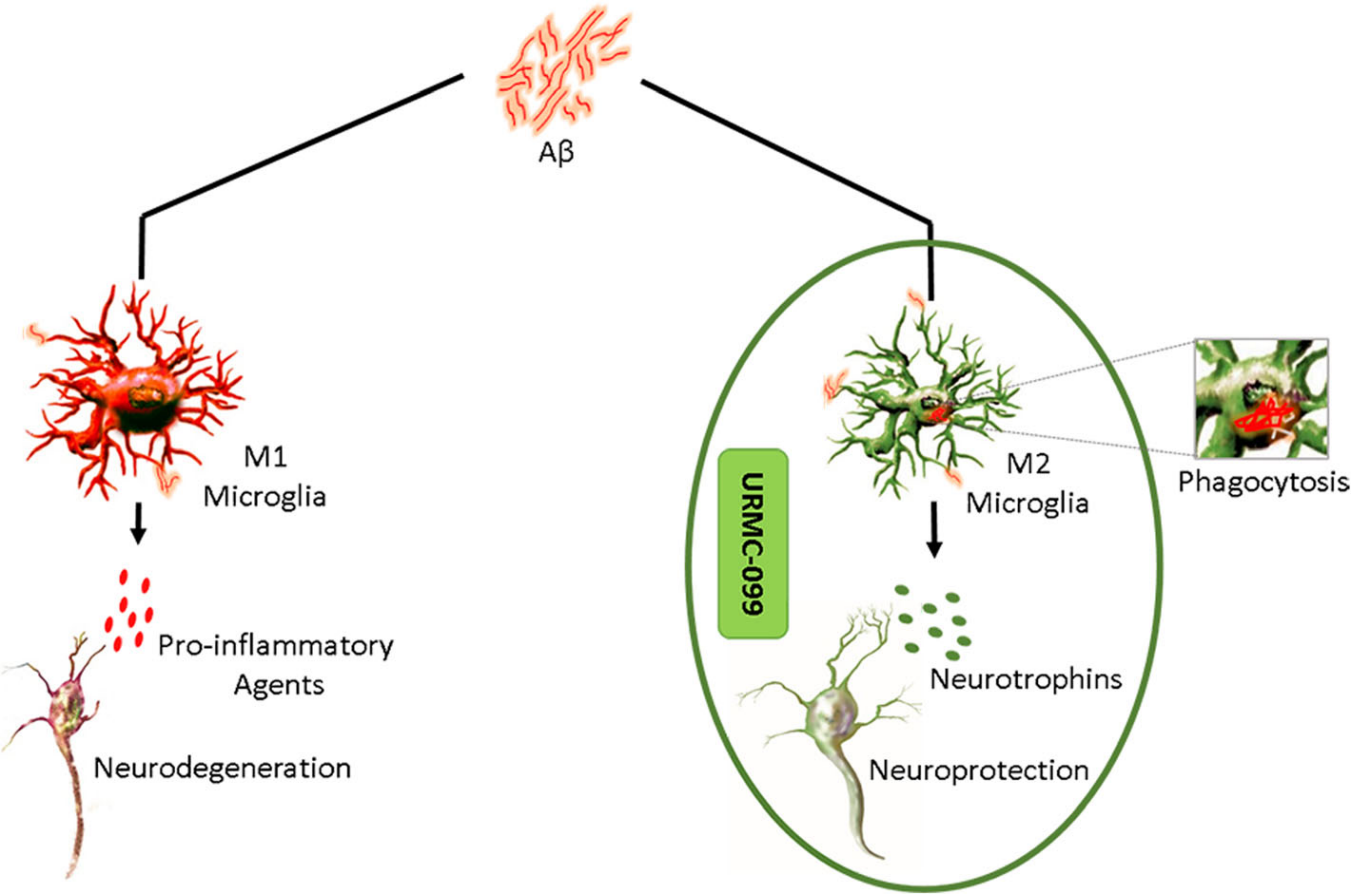
Prospective schematic diagram of URMC-099-mediated neuroprotective effects in APP/PS1 mice. Microglia acquires classical or alternative activation in Aβ vicinity. Classical microglial activation induces pro-inflammatory cytokines causing neuroinflammation and ultimately neurodegeneration developing AD-like conditions (left). URMC-099 facilitates alternative microglial activation, a more anti-inflammatory phenotype that phagocytes and degrades Aβ imparting neuroprotection in AD (right)

Indeed, our present APP/PS1 studies show that URMC-099 can reduce β-amyloidosis and microglial neuroinflammatory responses and improve synaptic integrity and hippocampal neurogenesis by affecting an anti-inflammatory microglial neurotrophic M2 phenotype. We also demonstrate that URMC-099 affects Aβ biogenesis, alters microglial morphology associated with cessation of a pro-inflammatory milieu, and elicits neuroprotective responses. Our findings in this AD model support prior studies demonstrating that URMC-099 can reverse microglial p38/JNK activation [18]. This is likely highly relevant as p38/JNK pathways are operative in the pathobiology of cerebral trauma, ischemia, AD, and Parkinson’s disease using animal models, as well as postmortem tissue samples [41, 65–67]. Inhibition of MLKs can abrogate activation of p38/JNK cascades in such pathological conditions to attenuate neuronal loss and reverse pathologic microglial activation [68, 69]. MLK3, a widely expressed member of MLK family, has also been implicated in microglial activation [7, 33]. MLK3 signaling in turn activates p38/JNK to negatively impact hippocampal integrity and function. Parallel phosphorylation of JNK substrates, c-Jun and Bcl-2, with activation of caspase-3 also directly promote neuronal apoptosis [70]. MLK/MAPK signaling is also involved in APP processing, and its inhibition favors production of the non-amyloidogenic α-secretase form of soluble APP (sAPPα) by inducing α-secretase activity, which in turn indirectly affects the production of pathogenic amyloid-induced through β- and γ-secretase activity [71–73]. Thus, MLK inhibitors promote the protective physiological roles of APP/sAPPα and α-secretase activity in AD. Herein, we show that URMC-099, a MLK3 inhibitor, inhibits the p38/JNK/MKK3/4 cascade, reduces β-amyloidosis, and restores microglial M2 and postsynaptic integrity assessed by the relative abundance of PSD-95. DG neurogenesis is also restored in APP/PS1 mice treated with URMC-099 [18]. Thus, treatment with URMC-099 plays a neuroprotective role during AD (Fig. 10) via multifactorial mechanisms.

In this and several of our prior works, it is clear that URMC-099 possesses multiple effects on the cell and in modulating the brain and peripheral tissue microenvironment [16, 18, 24]. The drug has been shown to facilitate the actions of long-acting nanoformulated antiretroviral drugs during HIV infection by its effects on autophagy. Notably, autophagy is also linked to microglial activities that include Aβ phagocytosis and clearance. In the current study, all of these potential therapeutic mechanisms of action of URMC-099 appear to converge with the drug acting as an immune modulator in control of phagolysosomal Aβ clearance and neuronal protection [18].

## Conclusion

Overall, we now demonstrate that URMC-099 facilitates Aβ clearance and protects impaired hippocampal neurogenesis. The multifaceted role of URMC-099 as a therapeutic agent makes it particularly attractive in affecting the control of complex disease course such as AD. Future research will ultimately determine if such long-term disease control can be sustained against the ongoing significant pathogenic destructive processes operative during neurodegenerative disease.

## Abbreviations

Abs: Antibodies
AD: Alzheimer’s disease
APP/PS1: Aβ precursor protein/presenilin-1
Aβ: Amyloid-beta
BBB: Blood-brain barrier
CNS: Central nervous system
Dcx: Doublecortin
DG: Dentate gyrus
ERK: Extracellular-signal-regulated kinase
HAND: HIV-associated neurocognitive disorders
HIV: Human immunodeficiency virus
i.p.: Intraperitoneal
IL: Interleukin
JNK-c: Jun amino-terminal kinase
Lamp1: Lysosomal-associated membrane protein 1
LRP1: Low-density lipoprotein receptor-related protein 1
MAPK: Mitogen-activated protein kinase
MLK3: Mixed lineage kinase type 3
MAPKKK: Mitogen-activated protein kinase kinase kinase
NFTs: Neurofibrillary tangles
NOS-2: Nitric oxide synthase-2
PAMP: Pathogen-associated microbial pattern
PBS: Phosphate-buffered saline
PEG400: Polyethylene glycol 400
PSD95: Postsynaptic density 95
RAGE: Receptor for advanced glycation end products
sAPPα: α-Secretase form of soluble APP
Tg: Transgenic
TGF: Transforming growth factor
TNF: Tumor necrosis factor
TS: Thioflavin-S
